# PG545 treatment reduces RRV-induced elevations of AST, ALT with secondary lymphoid organ alterations in C57BL/6 mice

**DOI:** 10.1371/journal.pone.0217998

**Published:** 2019-06-06

**Authors:** Aroon Supramaniam, Helle Bielefeldt-Ohmann, Penny A. Rudd, Julie Webster, Vito Ferro, Lara J. Herrero

**Affiliations:** 1 Institute for Glycomics, Griffith University, Gold Coast Campus, Southport, Queensland, Australia; 2 Australian Infectious Diseases Research Centre, School of Chemistry and Molecular Biosciences, The University of Queensland, St Lucia, Queensland, Australia; 3 School of Veterinary Science, The University of Queensland, Gatton Campus, Gatton, Queensland, Australia; National Institutes of Health, UNITED STATES

## Abstract

Recently the anti-viral effects of prophylactic treatment with the low-molecular-weight heparan sulfate mimetic PG545 in Ross River virus (RRV) infected mice were reported. We further investigated the related, transient pathophysiology of PG545 drug treatment in RRV-infected and mock-infected PG545-treated mice. PG545 treatment resulted in mild lethargy and piloerection, on days after the drug administration. Mice were treated with two or three doses of PG545 within a ten-day period and were subsequently culled at peak disease or at disease resolution. The treatment responses of the spleen and liver were assessed through histology, flow cytometry, gene arrays and serum biochemistry. Microscopy showed an expanded red pulp in the spleen following either two or three treatments with PG545. The red pulp expansion was further demonstrated by the proliferation of megakaryocytes and erythrocyte precursors within the spleen. In addition, flow cytometry and gene array analyses revealed a reduction of lymphocytes within the spleens of PG545-treated mice. Previously unreported, RRV-induced elevations of aspartate aminotransferase (AST) and alanine transaminase (ALT) enzymes and creatinine were also noted in the RRV-infected mice. However, PG545 only reduced AST and ALT levels but not the creatinine levels in infected mice during treatment. Mice treated with three doses of PG545 also showed hepatosplenomegaly and anaemia, which were reversed upon discontinuation of the treatment. In summary, this study demonstrates that dose and frequency related haemopoietic pathophysiology such as hepatosplenomegaly and anaemia, occurred in C57BL/6 mice treated with PG545. However, this effect was reversible once drug administration is terminated.

## Introduction

Australia is home to more than 70 arthropod-borne viruses that are mostly enzootic. However, a few exceptions, such as the Ross River virus (RRV) and Barmah Forest virus (BFV), can also infect humans and cause diseases [1]. Increased precipitation often leads to dramatic events such as extreme rainfall and non-tidal flooding [2]. These weather events in turn, enhance vector breeding and can exacerbate viral transmission to animal and human hosts, causing frequent, sporadic disease outbreaks [2]. RRV is an arthritogenic alphavirus in the *Togaviridae* family. It is transmitted either by the *Aedes* or *Culex* species of mosquitoes and causes notifiable diseases in Australia [3]. Infected individuals may be mildly febrile and experience debilitating peripheral polyarthralgia and myalgia. The musculoskeletal pain experienced can be either acute, or progress to chronic or recurrent pain, leading to significant morbidity [3]. In 2015, the largest epidemic in Australia reported a spike of 9,544 cases compared to the conventional annual incidence of around 5,000 cases [4, 5]. Then, in 2017, another 6,925 cases were reported [6]. Furthermore, the 2017 outbreak, also reported significant incidences of RRV disease (RRVD) in temperate regions such as Melbourne compared to the previous non-incidence in these cooler climate [7].

Current treatments such as NSAIDs only offer partial relief for patients with chronic or recurrent joint pain. Although, many studies are underway for better treatment options such as through the inhibition of specific immune cells, soluble host factors (macrophages, IL-6, macrophage inhibitory factor; MIF) or even through the development of preventative vaccines, only few studies have examined viral targeted treatments [8–11]. It was recently reported that PG545, (pixatimod), a cholestanol-conjugated, small molecule, heparan sulfate (HS) mimetic, effectively reduced acute pathological changes in RRV-infected mice and cells [12]. PG545 was initially developed as a dual heparanase and angiogenesis inhibitor and has progressed to clinical trials in cancer patients with advanced solid tumours following pre-clinical toxicology evaluations [13–15]. More recently, it has also been found to possess immunomodulatory activity [16]. PG545 exhibits potent ability to stimulate innate immune cells such as dendritic cells (DC) and natural killer cells (NK) while inhibiting tumour associated macrophages (TAM) and M2 macrophages against tumours in numerous cancer models in mice [15, 17]. PG545 was also described to have a direct inhibitory effect on RRV replication, as demonstrated by IC_50_ plaque assays (*in-vitro*) and reduction in viral loads in prophylactically treated mice (*in-vivo*). Furthermore, efficient amelioration of acute disease in RRV-PG545 treated mice, with marked improvements in virus infection related hind limb dysfunction, reduction in inflammatory cells and host soluble factors were reported with PG545 treatment [12].

To expand the scope of this work we extended the treatment to assess if a longer course of PG545 treatment could reduce musculoskeletal pathology in acute RRVD to baseline levels and alter disease kinetics by shortening recovery time. Furthermore, the treatment effect of PG545 and the potential for PG545-induced toxicity was evaluated by assessing secondary lymphoid organ responses to PG545 treatment in mock- and RRV-infected, C57BL/6 mice. PG545 treatment efficacies and related organ pathologies were investigated through two main types of treatment frequencies (standard; dual or prolonged; triple doses). Furthermore, the histopathological and immune responses of spleens, livers and overall blood parameters were also assessed. Results from these studies demonstrate that while both standard (previously reported) and prolonged PG545 treatment reduced RRV-induced joint dysfunction, prolonged PG545 treatment additionally caused transient anaemia and hepatosplenomegaly, which were quickly ameliorated after discontinuation of PG545. These haemopoietic imbalances were mild in the standard PG545 treatment. Additionally, we also report for the first time the presence of RRV-induced elevations of alanine transaminase (ALT) and aspartate aminostransferase (AST) and creatinine levels at peak disease. The absence of anaemia and hepatosplenomegaly in standard PG545-treated mice, suggests that PG545 has a significant but narrow therapeutic window for the prevention of RRV-induced clinical disease.

## Materials and methods

### Virus stocks and compound

The RRV-T48 strain, used to inoculate mice was generated using an infectious clone containing the full-length sequence (a kind gift from Richard Kuhn, Purdue University), as described elsewhere [8, 18]. Virus titres were quantified by titrating in Vero cells. PG545 was reconstituted in phosphate buffered saline (PBS, Sigma-Aldrich, Castle Hill, NSW, Australia) at 10 mg/ml, and stored at -20°C as previously described [14].

### Experimental animals and study design

C57BL/6 mice were obtained from the Animal Resources Centre (ARC, Murdoch, Western Australia). All animal experiments were conducted in strict accordance with the *Australian Code for the Care and Use of Animals for Scientific Purposes* and this study was approved by the Animal Ethics Committee of Griffith University under the permit; GLY/04/15. Mice aged 17–20 days, weighing 8–10 g were infected subcutaneously (s.c.) in the right thorax region with either 50 μl of 10^4^ plaque-forming units (PFU) of RRV, or mock-infected with a similar volume of 1X PBS. The standard PG545 treatment was administered as a dual dose intervention as previously published [12]. The prolonged regime was a triple dose with a preloading dose of 20 mg/kg at 1 day prior to the virus infection and subsequently, the maintenance doses of 10 mg/kg were given at 4 days post infection (dpi) and 9 dpi (S1A Fig). Mice were weight-matched and housed in gender specific cages with a maximum number of five animals per cage. They were exposed to 12-hour (hr) light/dark cycles and were allowed free access to standard rodent pellets and water. The animals were weighed prior to group allocation and every day until the experimental end-point. Additionally, infected animals were scored daily for hind-limb dysfunction and monitored for clinical signs of drug toxicity, such as shivers, weakness and piloerection as previously described [19].

### Specimen collection

Blood samples were collected into serum tubes by cardiac puncture (Greiner Bio-One, Maybachstraße, Frickenhausen, Germany). The coagulated blood was then centrifuged for 2 min at 3600 x g to obtain serum to be used for biochemical assays. A minimum of 10 μL of non-clotted blood was also collected into micro-cuvettes designed to enable haemoglobin readouts using the portable standard clinical blood haemometer (HemoCue 201+ System Haemoglobin Analyzer; Hemocue, Angelholm, Sweden). The organs were removed from each animal and examined to identify macroscopic alterations, such as colour or size. Then each organ was weighed. Additionally, the whole spleens and the left liver lobes were halved transversely and collected into vessels containing either 4% paraformaldehyde (PFA) (ProSciTech, Kirwan, QLD, Australia) fixative, Roswell Park Memorial Institute-1640 (RPMI-1640) media or RNAlater (both Sigma-Aldrich, Castle Hill, NSW, Australia).

### Biochemical analyses

Serum biochemical assays were conducted at IDEXX Laboratories (Brisbane), using approved standard operating procedures. Serum biochemical parameters, including albumin protein, alkaline phosphatase (ALP), glutamate dehydrogenase (GLDH), ALT, AST, total bilirubin, urea and creatinine, were determined by standard techniques using a clinical chemistry analyser.

### Flow cytometry

To characterise the leukocyte cell population, spleens were mechanically disrupted, to release single cell suspensions, then filtered through a 40 μm cell strainer (Sigma-Aldrich, Castle Hill, NSW, Australia). Red blood cells were lysed with ammonium-chloride-potassium (ACK) lysing buffer for 10 minute (min) and further filtered using a 40 μm cell strainer to remove cellular debris. Prior to antibody staining, the splenocytes were first incubated with anti-mouse CD16 / CD32 FC block (BD Pharmingen, clone 2.4G2; 1:100) for another 10 min to prevent non-specific background staining via Fc receptors. Then, splenocytes were immediately incubated with FITC-conjugated anti-mouse CD3 (BD Pharmingen, clone 145-2C11; 1:100) and PE-Cy7-conjugated anti-mouse CD45 (eBioscience, clone 30-F11; 1:100) antibodies diluted in FACS buffer (PBS with 2% foetal bovine serum (FBS, Invitrogen, Scoresby, VIC, Australia) for 45 min at 4°C. To terminate the labelling process, cells were washed in FACS buffer and re-suspended in propidium iodide (PI) 20 μg/mL (Sigma-Aldrich, Castle Hill, NSW, Australia) containing FACS buffer immediately before data acquisition using the CyanADP (Beckman Coulter, Lane Cove West, NSW, Australia). Dead cells and debris were excluded from the analysis. All analyses were done using the Kaluza Flow Analysis Software (Beckman Coulter, Lane Cove West, NSW, Australia).

### RNA isolation and complementary DNA conversion

Messenger RNA (mRNA) of spleen tissues were extracted utilising either commercially available PureLink RNA Mini Kit (Invitrogen, Scoresby, Vic, Australia) and the corresponding PureLink DNase Set (Invitrogen, Scoresby, Vic, Australia) or TRIzol Reagent (Invitrogen, Scoresby, Vic, Australia) according to the manufacturers' instructions. Spleens were collected in 1 mL RNAlater (Sigma-Aldrich, Castle Hill, NSW, Australia) for RNA extraction, then stored at 4°C for 48 hrs to enable penetration of RNAlater into the tissues. The spleens were then homogenised using a Tissuelyser II (Qiagen, Chadstone, VIC, Australia), centrifuged at 12,000 × g, 5 min, 4°C and the tissue homogenate was transferred to fresh tubes. Subsequently, RNA extractions were performed on the homogenates according to manufacturers’ instructions. Post-extraction the concentration and purity of the total RNA samples were measured using the NanoDrop ND-1000 spectrophotometer (NanoDrop Technologies Inc., Wilmington, USA). To prepare templates for gene expression studies, 1 μg of RNA was reverse transcribed to 50 ng/μL of complementary DNA (cDNA) using the iScript cDNA synthesis Kit (Bio-Rad, Gladesville, NSW, Australia).

### Quantitative real-time polymerase chain reaction (qRT PCR)

qRT PCR was performed using SsoAdvanced Universal SYBR Green Supermix either pipetted into individual wells of primer pre-plated, 96 panel haemopoiesis PrimePCR Assays (Bio-Rad, Gladesville, NSW, Australia) or with published primer sequences (Table 1). All qRT PCR runs were conducted using CFX96 Touch Real-Time PCR system using a standard three-step melt program; activation at 95°C for 2 mins, denaturation at 95°C for 5 s (× 40 cycles), annealing at 60°C for 30 s (x 40 cycles) and melt curve at 65 to 95°C at 0.5°C increments at 5 s per step. The difference in mRNA levels of each gene of interest between the groups was calculated as a relative fold change between treatment groups and mock-PBS group, using the ΔΔCt method. Briefly, ΔΔCt = ΔCt (RRV-infected)–ΔCt (Mock PBS) where ΔCt = Ct (gene of interest)–Ct (HPRT1). The final fold change for each gene was then, calculated as 2−ΔΔCt.

**Table 1:** Primer sequences.

Primer	Sequences	References
IL -10	(F) 5′-ATTTGAATTCCCTGGGTGAGAAG-3′ (R) 5′-CACAGGGGAGAAATCGATGACA-3′	[20]
CD 4+	(F) 5-TCC TTC CCA CTC AAC TTT GC-3 (R) 5-AAG CGA GAC CTG GGG TAT CT-3	[21]
CD 8α+	(F) 5-GCT CAG TCA TCA GCA ACT CG-3 (R) 5-ATC ACA GGC GAA GTC CAA TC-3	[21]
TfR1	(F) 5′-GAGGAACCAGACCGTTATGTTGT-3′ (R) 5′-CTTCGCCGCAACACCAG-3′	[22]
HBB-1	(F) 5’-CTGATTCTGTTGTGTTGACTTG-3’ (R) 5’-AGGTCTCCAAAGCTATCAAAGT-3’	[23]
GATA-1	(F) 5′-CACTCCCCAGTCTTTCAGGTGTA-3′ (R) 5′-GGTGAGCCCCCAGGAATT-3′	[24]
CSF2	(F) 5′-ATGCCTGTCACGTT-GAATGAAG-3′ (R) 5′-GCGGGTCTGCACACATGTTA-3′	[25]
RARA	(F) 5′- GCTTCCAGTCAGTGGTTACAGC -3′ (R) 5′- CAAAGCAAGGCTTGTAGATGCGG—3′	[26]
IKZF1	(F) 5′-TTGTGGCCGGAGCTATAAAC-3′ (R) 5′-TGCCATCTCGTTGTGGTTAG-3′	[27]
CD40LG	(F) 5’- GTGAGGAGATGAGAAGGCAA-3’ (R) 5’- CACTGTAGAACGGATGCTGC -3’	[28]

### Solid phase sandwich ELISA

The serum levels of IL-10 in the experimental mice, from the four treatment groups, were measured using a sandwich ELISA (DuoSet ELISA DY417-05; 1:3 dilution). Briefly, 100 μL of either standard or sample was added to each well, and the plate was taped and incubated for 2 hrs at room temperature (r.t). Then the liquid in each well then was decanted and washed three times with wash buffer (0.05% tween in PBS pH 7.4) and subsequently, 100 μL of a working solution of biotinylated detection antibody was added to each well. The plate was then covered and incubated for another 2 hrs at r.t. Each well then was washed with wash buffer three times before 100 μL of horseradish peroxidase (HRP)-conjugated working solution was added to each well, and the plate was covered and incubated for a further 20 min at r. t. Each well was washed with wash buffer three times, 100 μL of substrate was added to each well, and the plate was covered and incubated for a further 20 min at r.t. At experimental end-point, 50 μL of 2N HCL stop solution was added to each well to stop the reaction when the wells turned a medium shade of blue. The optical density (OD) value of each well was determined immediately using a microplate reader at a wavelength of 450 nm. Subsequently, the IL-10 concentrations were calculated using the standard curve.

### Tissue processing, staining and histopathological evaluations

For histopathological studies, tissues such as the kidneys, spleens and liver lobes were fixed in 4% PFA at 4°C for 24 hrs. Following fixation, the tissues were PBS rinsed for 2 hrs then stored in 70% ethanol, prior to the routine processing for paraffin embedding. Five μm sections were collected onto glass slides, deparaffinised using histosolv-X solvent (Trajan Scientific, Ringwood, Vic, Australia) and stained with either haematoxylin and eosin (H&E) or Leder (stains for chloroacetate esterase activity present in the granules of granulocytes) stains. The tissues from all groups were then critically evaluated by light microscopy for any evidence of histopathological changes by a veterinary pathologist blinded to treatments and infection status. Changes were scored as follows: grade 0 = within normal limits/no change, grade 1 = minimal change, grade 2 = mild change, grade 3 = moderate change, grade 4 = marked/severe change. Photomicrographs of representative slides were imaged using the Aperio AT2 slide scanner (Leica Biosystems, USA).

### Statistical analyses

All statistical analyses were performed using GraphPad Prism version 6 software (GraphPad Software Inc). Clinical differences between the mock-treated group and the PG545-treated group were assessed using the Mann-Whitney *U* test, while weight gain, fold change haematological (haemoglobin) levels, absolute / relative organ weight, biochemical indices and histopathology scores between the treated and control groups, were either analysed using2-way analysis of variance (ANOVA) with Bonferroni post-test, or were compared using one-way ANOVA, followed by Dunnett’s post-test to determine the level of significance when compared against mock-PBS group alone. All data are presented as mean ± standard error of mean (SEM) and any *P* values less than 0.05 were considered statistically significant.

## Results

### Prolonged PG545 treatment does not completely ameliorate RRVD in mice

To evaluate the drug efficacy, 17–20-day old C57BL/6 mice were randomly allocated into four groups of mock-infected PBS-treated (mock-PBS), mock-infected PG545-treated (mock-PG545), RRV-infected PBS-treated (RRV-PBS) and RRV-infected PG545-treated (RRV-PG545) mice. These groups of animals were then further separated according to their assigned treatment of dual (standard) or triple (prolonged) PG545 doses (S1A Fig). Mice were weighed daily until experimental endpoint as previously reported [12] (S1B and S1D Fig).

Prolonged PG545 treatment was investigated primarily to determine if acute disease could be completely ameliorated and/or the kinetics of disease resolution could be altered, as well as to assess dose dependent organ toxicity in PG545 treatment to mice (S1E Fig).

Both the standard and prolonged, PG545-treated RRV-infected mice had similar reductions in disease kinetics (S1C and S1D Fig) and in RRV-induced weight loss (S1B and S1D Fig) demonstrating that the increase in treatment frequency had no added beneficial effect in terms of reduction of acute clinical disease or improvement to disease resolution in infected mice.

Neither the dual nor triple administrations of PG545 produced any severe signs of toxicity in animals. Mild overt signs included lethargy, piloerection and weight loss. Further, prophylactic triple dosed, PG545 treated mouse cohorts, also showed matching weight to their mock-PBS littermates by clinical disease recovery phase at 22 dpi indicating any effect of PG545 on weight was transient.

### PG545 treatment induces splenomegaly through extramedullary haematopoiesis and white pulp atrophy

**Fig 1:**
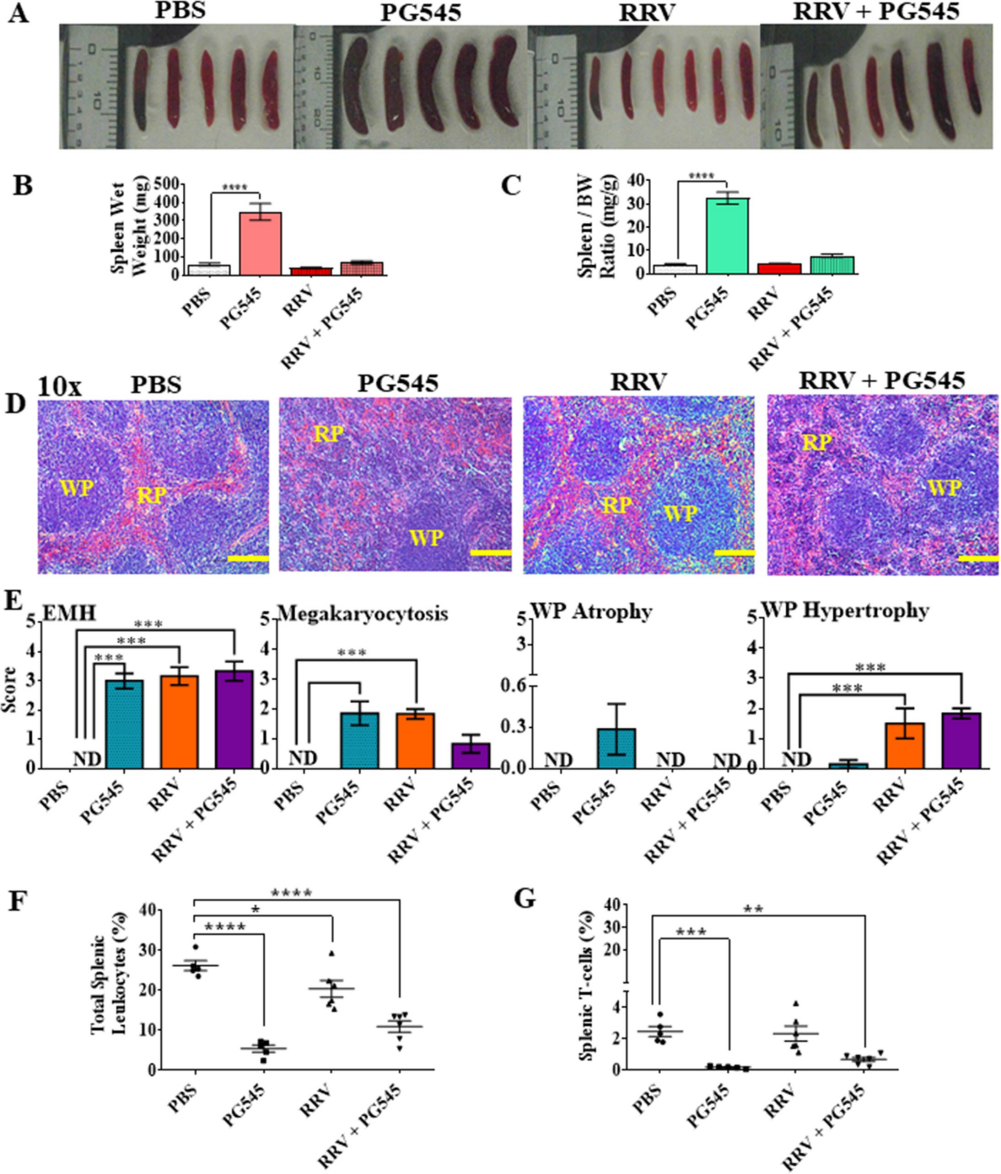
PG545 treatment induces extramedullary haematopoiesis in red pulp and white pulp atrophy. 17-20-days-old C57BL/6 mice were infected s.c. with 10^4^ PFU/50μl RRV or PBS alone and received s.c. injections of PG545 or mock-treatment with PBS on -1 and 4dpi (A) Representative spleens per treatment group were imaged. (B) Actual spleen weight and (C) relative organ/body weight ratios of mice from corresponding treatment groups were plotted. (D) Representative histomorphology of spleens sections stained with H&E (Scale bar; 10 x = 200 μm). (E) Spleen sections were assessed and scored according to level of EMH, megakaryocytosis, white pulp atrophy or hypertrophy. (F) Graphical representation of percentages of CD45+ infiltrating leukocytes and (G) CD3+ T-cells isolated from murine spleens per experimental group. Each data point represents the mean ± SEM (n = 5–7). RP, red pulp; WP, white pulp; EMH, extramedullary haematopoiesis; PBS, mock-infected PBS control; PG545, mock-infected PG545-treated; RRV, RRV-infected PBS-treated; RRV + PG545, RRV-infected PG545-treated.

To investigate if the PG545 treatment causes lymphoid organ related pathology, spleens were examined macroscopically at 10 dpi across all four groups. The spleens of mice from mock-PG545 treated groups showed gross abnormality in size when compared to the mock-PBS group (Fig 1A). Spleens of the mock-PG545 group were visually larger than mock-PBS spleens (Fig 1A). Moreover, both the absolute weight and spleen weight relative to the body weight of mock-PG545 mice was approximately three times higher (**** = P<0.*0001*) than mock-PBS, RRV-PBS and RRV-PG545 mice groups (Fig 1B and 1C). Interestingly we also noted some mild spleen enlargement in the RRV-PG545 mice group, although this was found to be not significant.

The spleen has both haemopoietic and immunologic functions and therefore, both the red and white pulp were evaluated for pathological changes [29]. Histopathological analysis of the spleen sections showed prominent extramedullary haematopoiesis (EMH) with highly vascular red pulp and increased numbers of megakaryocytes (megakaryocytosis) in all groups except for mock-PBS group (Fig 1D and 1E). Interestingly, while the spleens of mock-PG545 mice showed mild white pulp atrophy (Fig 1D and 1E), RRV-PBS and RRV-PG545 treated spleens showed mild hypertrophy of the white pulp suggesting that RRV infection results in expansion of total leukocyte numbers. Flow cytometry was used to characterise the effect of RRV infection and treatment on immune-cells. Data showed a significant reduction in percentages of CD45+ total leukocytes and CD3+ T-cells in both mock-PG545 (CD45+, **** = P<0.*0001*; CD3+, *** = P<0.*001*) and RRV-PG545 groups (CD45+, **** = P<0.*0001*; CD3+, ** = P<0.*01*) (Fig 1F and 1G) compared to mock-PBS group. Moreover, the percentages of CD45+ total leukocytes were also significantly reduced in the RRV-PBS group at * = P<0.*05* in comparison to the mock-PBS group. Overall, the data shows that the splenomegaly in PG545-treated mice was a red pulp-based expansion with atrophy of the white pulp as reflected both morphologically and as reduced numbers of CD45+ and CD3+ cells.

### PG545 treatment induces granulocyte recruitment in spleens of RRV-infected mice

**Fig 2:**
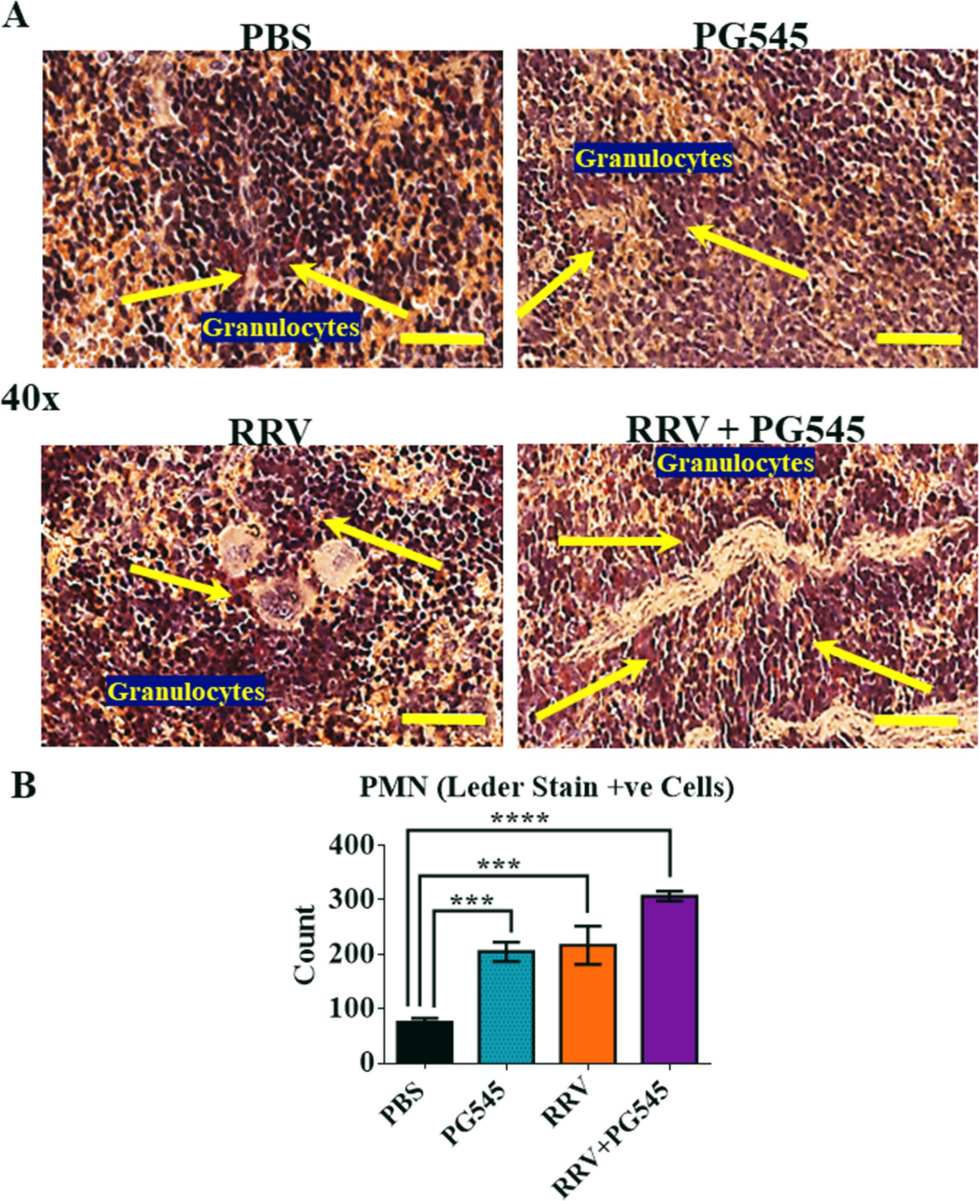
PG545 treatment of RRV-infected mice causes an increased recruitment of granulocytes. 17–20 -day-old C57BL/6 mice were infected s.c. with 10^4^ PFU RRV or PBS alone on day 0. Then, infected mice were either or mock treated with PBS, or with PG545 on days −1, 4 and 9 dpi. For histological analysis, sections of spleen tissue were stained with Leder; granulocytes stained copper brown (yellow arrows). (A) Images are representative of at least 5 mice per group. (Scale bar, 40× = 50 **μ**m). (B) Positively stained PMN cells were quantified using 10 non-overlapping, randomly selected areas, mainly but not exclusively comprising red pulp and graphed across the groups. PBS, mock-infected PBS control; PG545, mock-infected PG545-treated; RRV, RRV-infected PBS-treated; RRV + PG545, RRV-infected PG545-treated.

To further evaluate the spleen pathology, sections were stained with Leder then scored for immune-cell infiltrate using a double-blind research design. Leder sections showed presence of polynuclear neutrophils (PMN) in all groups. The PMN enumeration from both the red and white pulps (Fig 2B), showed greatest abundance of cells in in RRV-PG545 (**** = P<0.*0001)* > RRV-PBS (*** = P<0.*001)* and lowest frequencies in the spleens of mock-PBS mice (Fig 2A and 2B). Overall, the data shows that the splenomegaly in PG545 treated mice was a red pulp-based expansion with atrophy of the white pulp as reflected both morphologically and as reduced numbers of CD45+ and CD3+ cells (Fig 1F and 1G).

### PG545 treatment protects from RRV-induced elevations of enzymes AST and ALT

**Fig 3:**
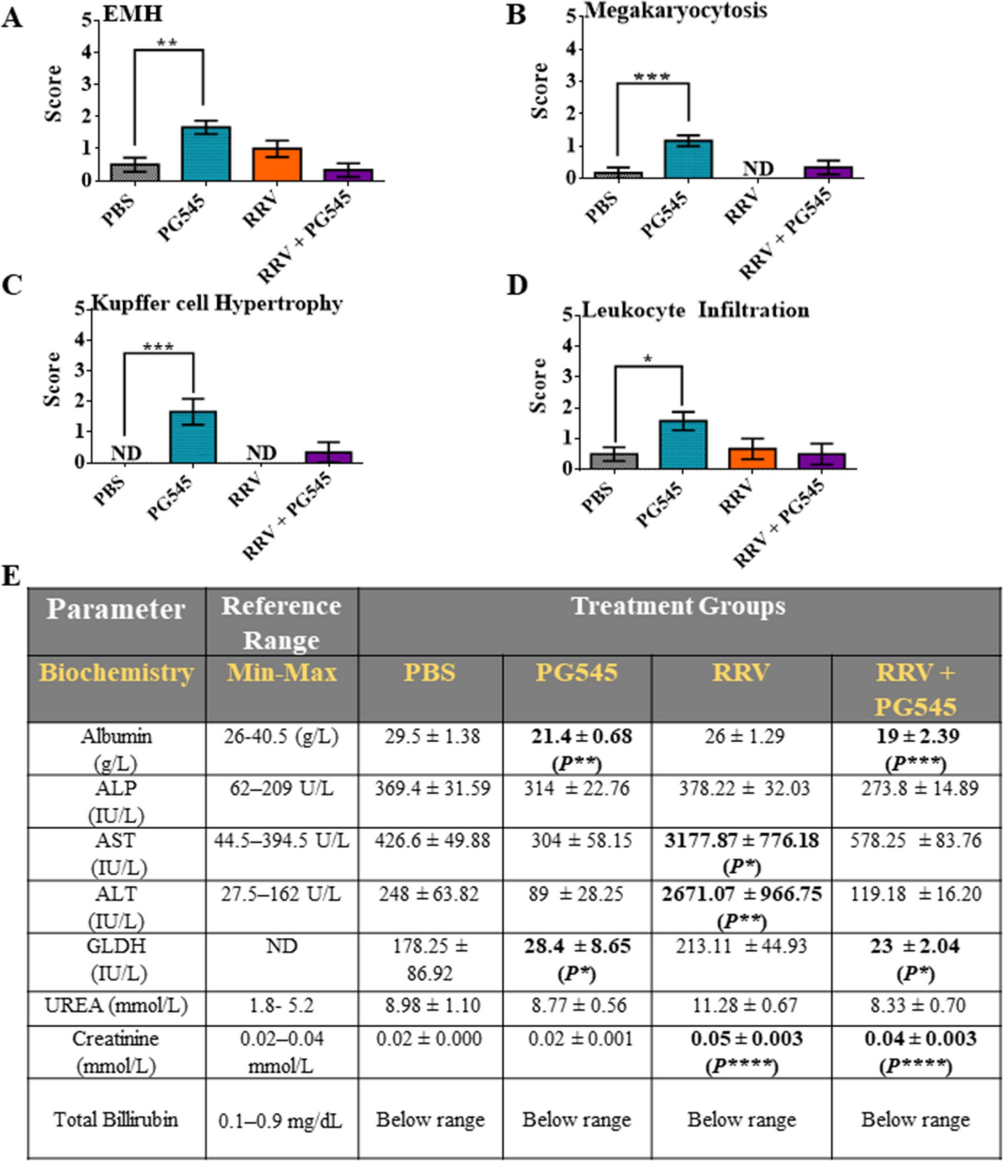
PG545 treatment caused mild changes in liver parenchyma with protection against RRV-induced elevation of AST and ALT enzymes. 17–20-day-old C57BL/6 mice were infected s.c. with 10^4^ PFU RRV or PBS alone on day 0. Then, infected mice were either or mock-treated with PBS, or with PG545 on days −1 and 4 dpi. Mice were culled at 10 dpi, with liver and serum harvested. (A-D) Liver sections were H&E stained, assessed and scored according to level of EMH, megakaryocytosis, leukocyte infiltration and Kupffer cell hypertrophy. (E) Serum from mice were assayed for selected liver specific biochemistries. All data represent the mean results ± SEM for at least 5 mice per group. PBS, mock-infected PBS control; PG545, mock-infected PG545-treated; RRV, RRV-infected PBS-treated; RRV+PG545, RRV-infected PG545-treated; EMH, extramedullary haematopoiesis; ALP, alkaline phosphatase; AST, aspartate aminotransferase; ALT, alanine aminotransferase; GLDH, Glutamate dehydrogenase.

To investigate if PG545 treatment causes any drug-induced liver or kidney pathology, liver and kidney sections were stained with H&E, then scored for immune-cell infiltrate or tubular injury respectively, using a double-blind research design. H&E liver sections showed minimal to mild EMH across all groups (Figs 3A and S2). RRV-PBS treated livers had minimal to mild EMH mainly comprised of myeloid proliferation and scattered megakaryocytes (Figs 3A, 3B and S2). Mock-PG545 treated livers had EMH with additional mild leukocyte infiltrations and activated Kupffer cells throughout the parenchyma (Figs 3A, 3C, 3D and S2). Although all parts of nephrons and vasculature of the cortex were assessed of the kidney sections, only mild tubular dilation with increased luminal content, and minimal to mild glomerular hypertrophy were found in both RRV-PBS (* = P<0.*05*) and RRV- PG545 kidneys s (* = P<0.*05*) compared to mock-PBS kidneys (S3 Fig).

Drug induced hepatotoxicity was investigated by assaying a panel of biochemical parameters across all groups as shown in Fig 3E. Unexpectedly, RRV infection caused a significant increase in both serum ALT (** = P<0.*01*) and AST (* = P<0.*05*), with no changes noted in serum albumin and GLDH levels (Fig 3E). However, after treatment with PG545, serum ALT and AST levels in RRV-infected mice returned to mock-PBS levels, indicating that the standard PG545 treatment protected against the RRV-induced elevations of tissue enzymes. Furthermore, PG545 treatment lowered the GLDH levels significantly in both mock-PG545 (* = P<0.*05*) and RRV-PG545 (* = P <0.*05*) mice, once again suggesting that PG545 may be tissue protective and therefore, reduce the release of parenchymal enzymes. Serum albumin levels were also significantly lower in both mock-PG545 (** = P<0.*01*) and RRV-PG545 (*** = P<0.*001*). In addition, RRV infection significantly increased serum creatinine levels in both RRV-PBS and RRV-PG545 (**** = P<0.*0001*). However, unlike the ALT and AST enzymes, PG545 treatment could not return the creatinine levels to mock-PBS levels, indicative of potential muscle and kidney damage in the course of RRV infection. Although, total bilirubin was analysed in all treatment groups, it was too low to be detected in any one treatment group.

### Prolonged PG545 treatment regime causes reversible hepatosplenomegaly and anaemia

**Fig 4:**
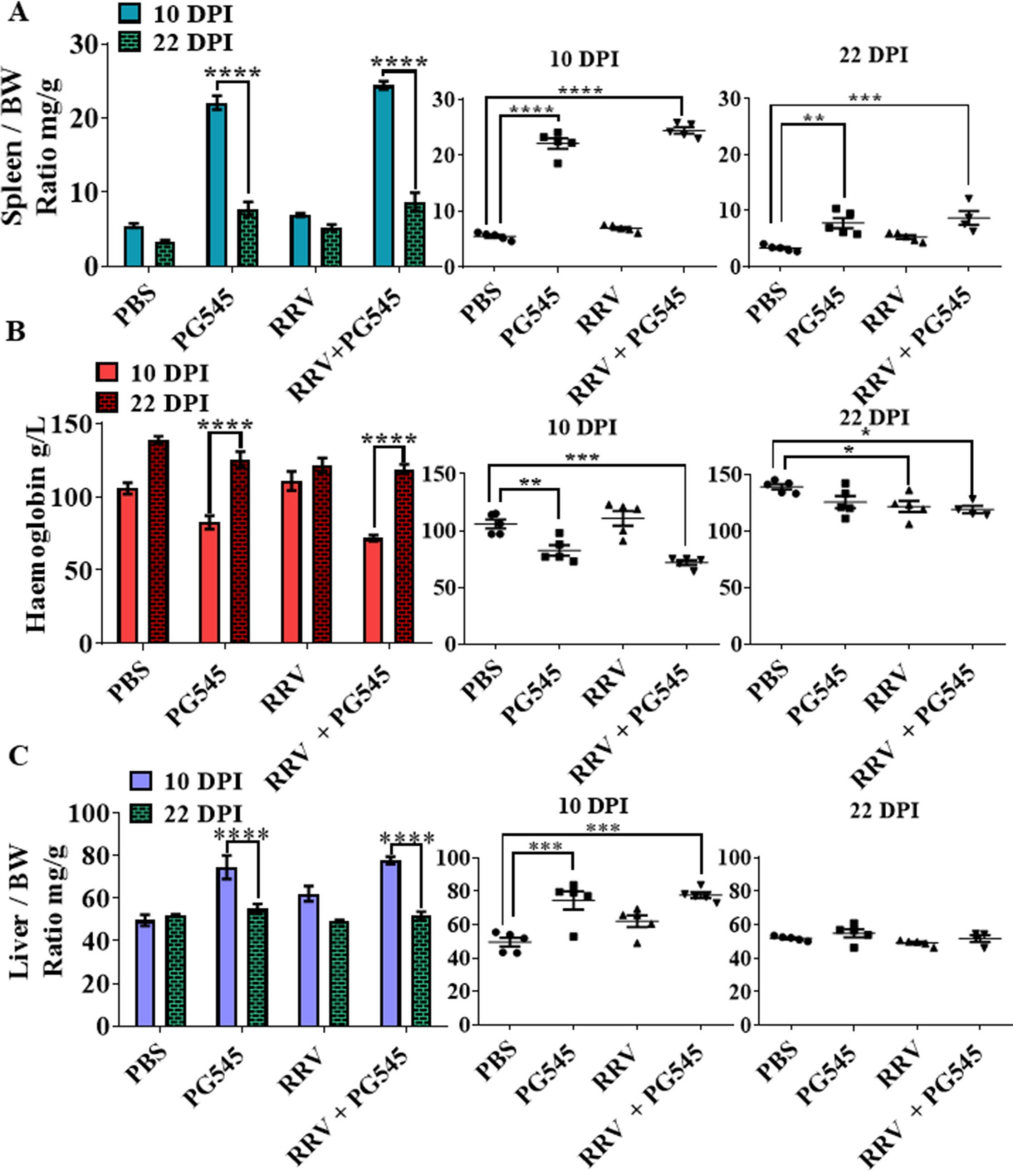
Prolonged PG545 at triple doses causes transient hepatosplenomegaly and anaemia in treated mice. 17-20-day-old C57BL/6 mice were infected (s.c.) with 10^4^ PFU/50μL RRV or PBS alone on day 0 and received s.c. injections of PG545 or PBS diluents from on −1, 4 and 9 dpi. (A, C) Mice were sacrificed at 10 dpi and 22 dpi and relative organ/body weight ratios of spleen and liver were graphed. (B) Haemoglobin levels of 10 dpi and 22 dpi mice were recorded using a portable standard clinical blood haemometer as described in the materials and methods section. All data represent the mean results ± SEM for at least 5 mice per group. PBS, mock-infected PBS control; PG545, mock-infected PG545-treated; RRV, RRV-infected PBS-treated; RRV + PG545, RRV-infected PG545-treated.

To investigate the extent of organ pathological changes in the prolonged PG545 treatment regime, spleen and liver weights relative to body weight were calculated. As seen in the standard regimen, the prolonged treatment resulted in mock-PG545 spleens that were significantly larger than the mock-PBS (**** = P<0.*0001*) (Fig 4A). Interestingly, the relative spleen weights were equally high in RRV-PG545 mice and mock-PG545 mice, and this was statistically significant in comparison to the spleens of mock-PBS mice (Fig 4A). To determine if the drug-induced splenomegaly could be a transient effect, spleens of mice were excised and weighed at 22 dpi, when compound administration had been discontinued 13 days prior, at 9 dpi. While the spleens in both mock-PG545 (** = P<0.*01*) and RRV-PG545 (*** = P<0.*001*) were still significantly larger than mock-PBS at 22 dpi (Fig 4A), the spleen size relative to body weight had reduced to half of that in 10 dpi (Fig 4A) (**** = P<0.*001*). This suggests that the splenomegaly was transient and reversible.

Given the expansion of the red pulp and EMH was noted in the standard PG545 treatment (Fig 1D and 1E), haemoglobin levels were also measured for signs of anaemia at necropsy (Fig 4B). It was found that haemoglobin levels of mice were inversely proportional to their spleen sizes (Fig 4B). Mock-PG545 and RRV-PG545 mice were found to be anaemic with haemoglobin levels under 100 mg/L and significantly lower than mock-PBS at 10 dpi (Fig 4B). By 22 dpi, mice were no longer anaemic (Fig 4B), however haemoglobin levels in RRV-PG545 (* = *P<0*.*05*) mice were still significantly lower relative to the 22 dpi mock-PBS control mice. Overall, haemoglobin levels at 22 dpi had returned to baseline suggesting that mice recovered from the compound-induced anaemia, when the treatment was discontinued.

Consistent with the spleens, there was a significant increase in liver mass and mass relative to body weight at 10 dpi. The ratio of liver mass relative to body weight for both mock-PG545 (*** = *P<0*.*001*) and RRV-PG545 (*** = *P<0*.*001*) were significantly larger, at approximately 1.5 times, the relative weight of livers from the mock-PBS group (Fig 4C). However, signs of hepatomegaly were completely reversed, and the liver/body weight ratio returned to levels comparable to those of mock-PBS mice at 22 dpi (Fig 4C).

### Prolonged PG545 treatment causes reversible white pulp atrophy in mouse spleens

**Fig 5:**
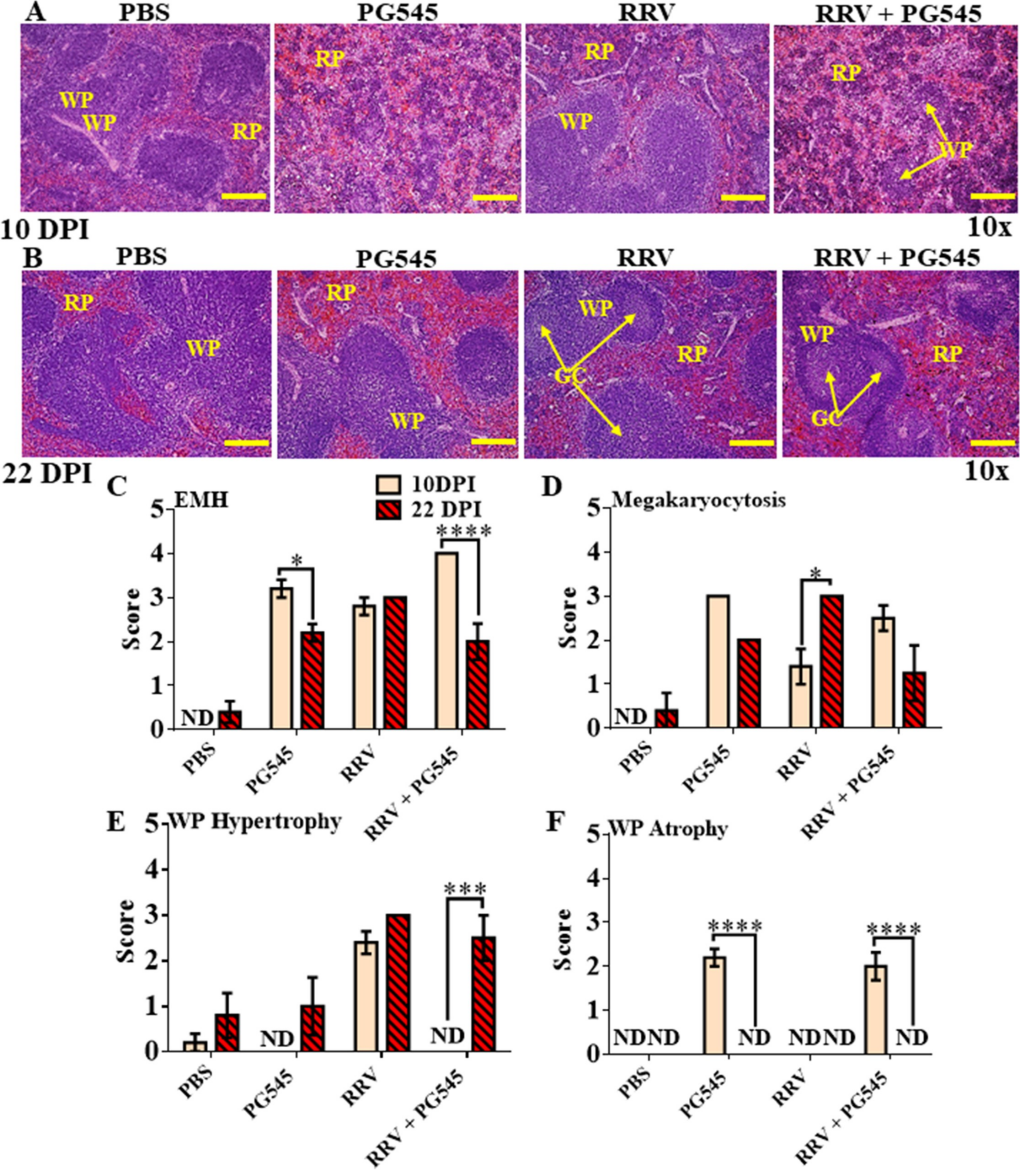
Prolonged PG545 treatment at triple doses causes white pulp atrophy in mice spleens that is completely reversed by 22 dpi. 17-20-day-old C57BL/6 mice were infected s.c. with 10^4^ PFU or PBS alone on day 0. Then, infected mice were either mock treated with PBS, or with PG545 on days −1, 4 and 9 dpi. (A-B) For histological analysis, paraffin embedded spleen tissues were stained with H&E. (C-F) Spleen sections were assessed and scored according to level of EMH, megakaryocytosis, white pulp atrophy or hypertrophy for both 10 dpi and 22 dpi time points. All data and images represent the mean results ± SEM for at least 5 mice per group (Scale bar; 10× = 200**μ**m). RP, red pulp; WP, white pulp; GC, germinal centre; PBS, mock-infected PBS control; PG545, mock-infected PG545-treated; RRV, RRV-infected PBS-treated; RRV + PG545, RRV-infected PG545-treated.

Prolonged PG545 treatment also resulted in marked EMH with cells from the erythroid and myeloid lineages as well as megakaryocytes present in the splenic red pulp of all three drug-treated groups (Fig 5A, 5C and 5D). Mock-PBS mice had a moderately developed white pulp with multiple lymphoid follicles, some of which contained germinal centres (i.e., secondary follicles), indicating a normal active spleen (Fig 5A). RRV-PBS mice presented with mild to moderate hypertrophy of the white pulp (Fig 5A and 5C). In contrast, the changes in spleen morphology in both mock-PG545 and RRV-PG545 treated mice presented as mild to moderate atrophy of the white pulp, which was disrupted/dispersed by the EMH in the red pulp (Fig 5A, 5C and 5F).

By 22 dpi, mock-PBS mice presented with normal spleen morphology, including well-developed white pulps with several secondary follicles (germinal centres) (Fig 5B). Furthermore, the mock-PG545, RRV-PBS and RRV-PG545 mice, now presented with moderate hypertrophy of the white pulp in addition to also having many secondary follicles (Fig 5B and 5E). Additionally, moderate increase in EMH in the subcapsular zone and extending into the central red pulp with the involvement of all cell lineages was also noted across all groups other than the mock-PBS mice (Fig 5B and 5C).

### Prolonged PG545 treatment regime temporarily dysregulates haemopoietic pathways in the spleen

**Fig 6:**
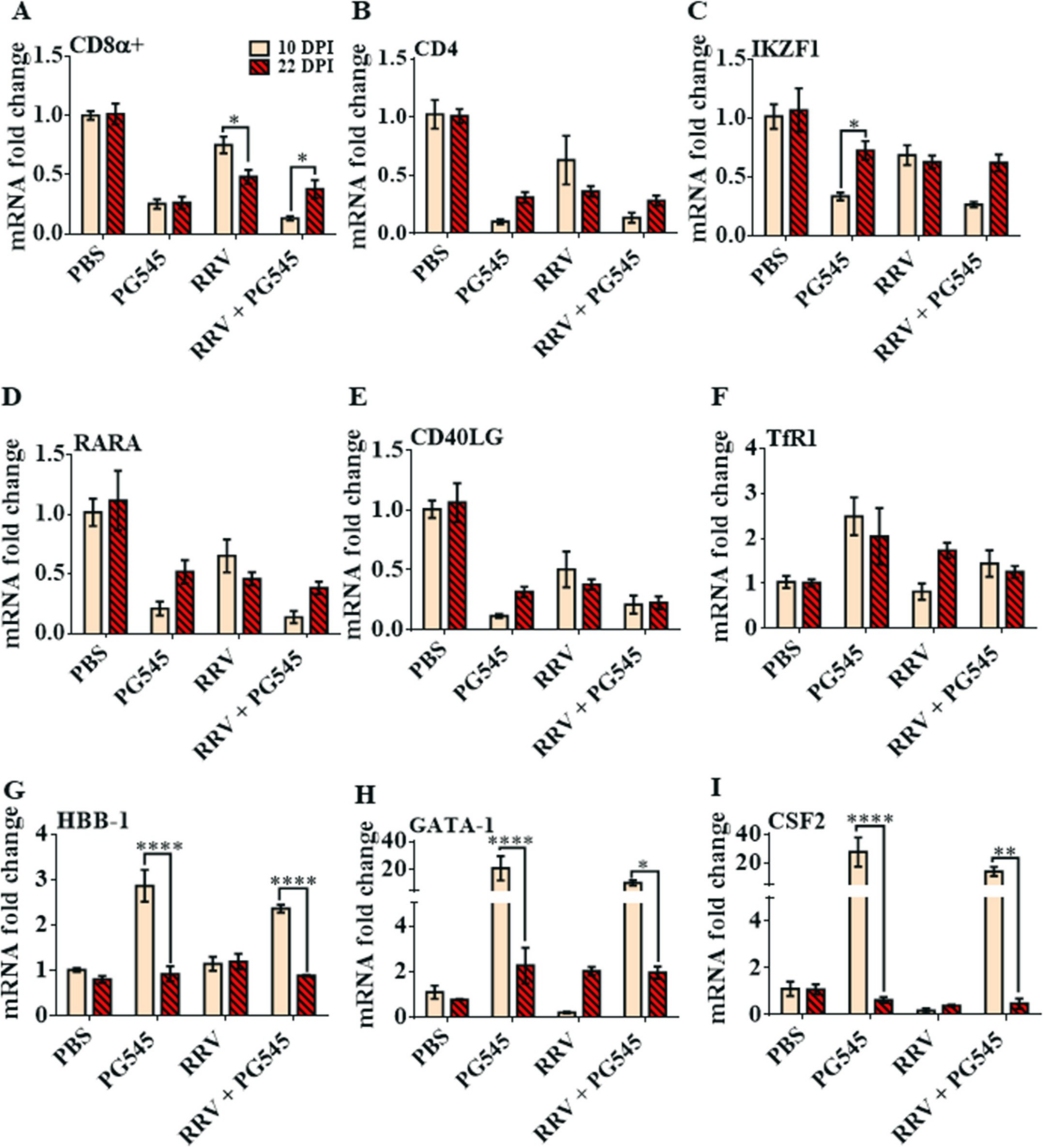
Prolonged PG545 treatment at triple doses induces reversible haemopoietic imbalances in treated spleens at 10dpi. 17-20-day-old C57BL/6 mice were infected (s.c.) with 10^4^ PFU/50 μl RRV or PBS alone on day 0 and received s.c. injections of PG545 or PBS diluents from −1, 4 and 9 dpi. (A-I) Mice were sacrificed on 10 or 22 dpi and spleens were homogenised, and RNA extracted to analyse haemopoietic influencing soluble host factors, transcription factors or immunocyte expression via RT-qPCR. (A-I) The y-axis is the final calibrated genomic material expression graphed as fold shifts and the x-axis represents the treatment groups; PBS = Mock-PBS control, PG545 = Mock-infected PG545- treated, RRV = RRV-infected PBS-treated, RRV + PG545 = RRV-infected PG545-treated. A-E) Shows specific genes that are reversibly down-regulated in mock- PG545 and PG545 + RRV groups at 10 dpi (versus 22 dpi). FI) Shows specific genes that are reversibly up-regulated in mock- PG545 and PG545 + RRV groups at 10 dpi (versus 22 dpi). Values are expressed as mean fold change in expression ± SEM for 4–5 mice each group.

To further characterise the haemopoietic influences of both PG545 drug treatment and RRV infection in the spleens of mice at peak disease, a panel of haemopoietic influencing genes were analysed through RT-qPCR. Corroborating the reduction in percentages of CD45+ and CD3+ cells, as described previously (Fig 1F and 1G), mRNA expression analysis revealed that both cytotoxic CD8a+ T-cells and helper CD4+ T-cells were down-regulated in mock-PG545 and RRV-PG545 spleens relative to the mock-PBS control spleens at 10dpi (Fig 6A and 6B). Additionally, CD40 ligand (CD40LG), transcription factor IKAROS family zinc finger 1 (IKZF1) and RARA were all also reduced in mock-PG545 and RRV-PG545 mice spleens relative to mock-PBS mice spleens at 10dpi (Fig 6C–6E). Interestingly by 22 dpi, the expression levels of cytotoxic CD8a+ T-cells, helper CD4+ T-cells, IKZF1, CD40LG and RARA in mock-PG545 and RRV-PG545 spleens were comparable to the RRV-PBS spleens and were no longer compromised or down-regulated.

Furthermore, mock-PG545 and RRV-PG545 mice spleens also demonstrated significant increases in transferrin receptors (TfR1), haemoglobin subunit beta chain (HBB-1), GATA binding protein 1 (GATA-1) and colony-stimulating factor 2 (CSF2) at 10 dpi (Fig 6H–6I). As seen in Fig 6A–6E, these haemopoietic up-regulations were transient and were reversed by 22 dpi. In particular, significant decreases were noted in mock-PG545 and RRV-PG545 groups between 10 dpi and 22 dpi of GATA-1 (mock-PG545, **** = P<0.*0001*; RRV-PG5454, * = P<0.*05*), CSF2 (mock-PG545, **** = P<0.*0001; RRV-PG545*, ** = P<0.*01)* and HBB-1 (mock-PG545, **** = P<0.*0001; RRV-PG545*, *** = P<0.*001)* (Fig 6F–6I). Overall, the gene expression data confirms the inverse relationship of the red and white pulps within the spleen at prolonged PG545 treatment, where the treatment also causes expansion of the red pulp and atrophy of the white pulp.

### Prolonged PG545 treatment causes elevations of genomic and translated levels of IL-10 in mice at 10dpi

**Fig 7:**
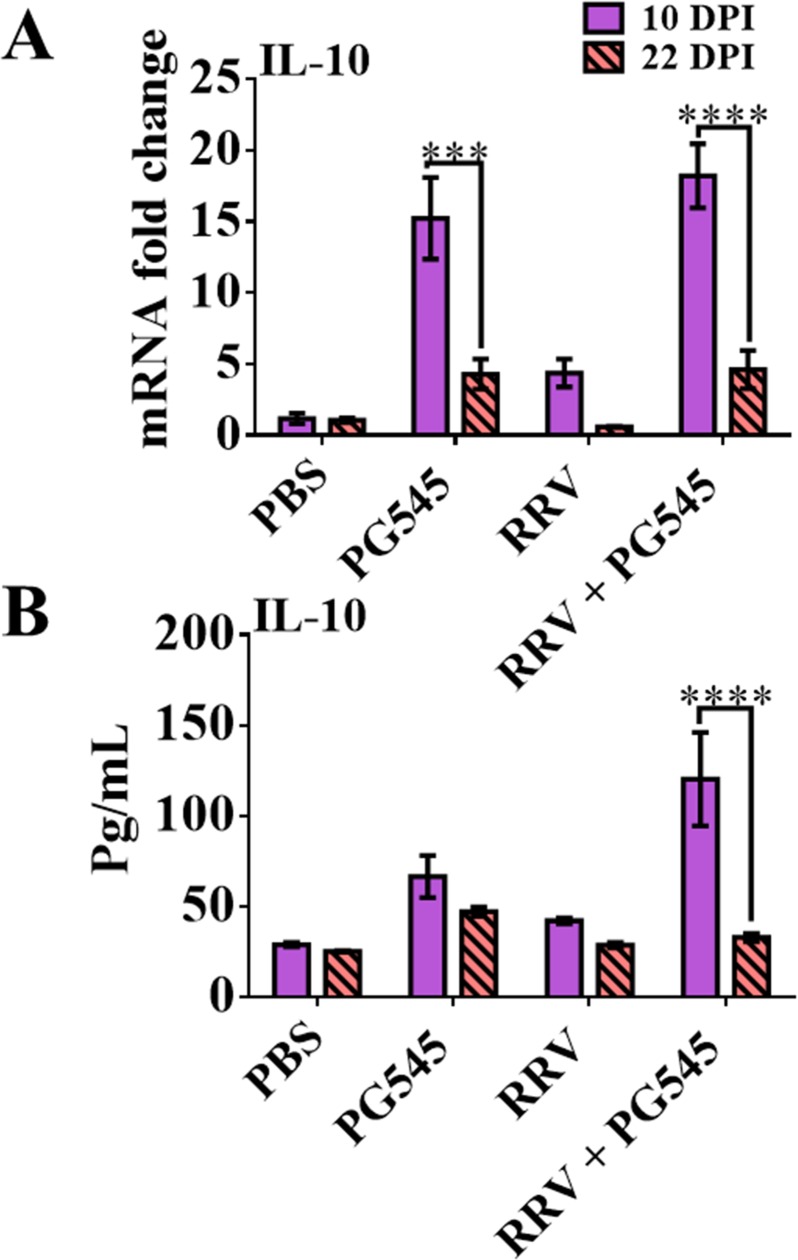
Prolonged PG545 treatment causes genomic and protein IL-10 elevation in mice at 10dpi. 17–20-day-old C57BL/6 mice were infected s.c. with 10^4^ PFU RRV or PBS alone on day 0. Then infected mice were either mock treated with PBS or with PG545 on −1, 4 and 9 dpi and subsequently, sacrificed at 10 dpi and 22 dpi. (A) For RT-qPCR analysis, spleens were homogenised, and RNA extracted to analyse soluble host factor IL-10 expression levels. The y-axis is the final calibrated genomic material expression graphed as fold shifts and the x-axis represents the relevant treatment groups (B) Serum samples were assayed for IL-10 protein levels using developmental ELISA kit. OD readings were extrapolated against a set of 7 standards to determine protein concentrations in pg/mg. All data represent the mean results ± SEM for at least 5 mice per group. PBS, mock-infected PBS control; PG545, mock-infected PG545-treated; RRV, RRV-infected PBS-treated; RRV + PG545, RRV-infected PG545-treated.

To further assess the potential role of IL-10 with regards to prolonged PG545 treatment, levels of IL-10 were measured in both serum and spleen (Fig 7). Data showed that IL-10 levels were significantly up-regulated both systemically in the serum and locally in the spleens of mock-PG545 mice, (*** = P<0.*001)* and RRV-PG545 mice (**** = P<0.*0001)*.

### Prolonged PG545 causes transient EMH and moderate inflammation in liver

**Fig 8:**
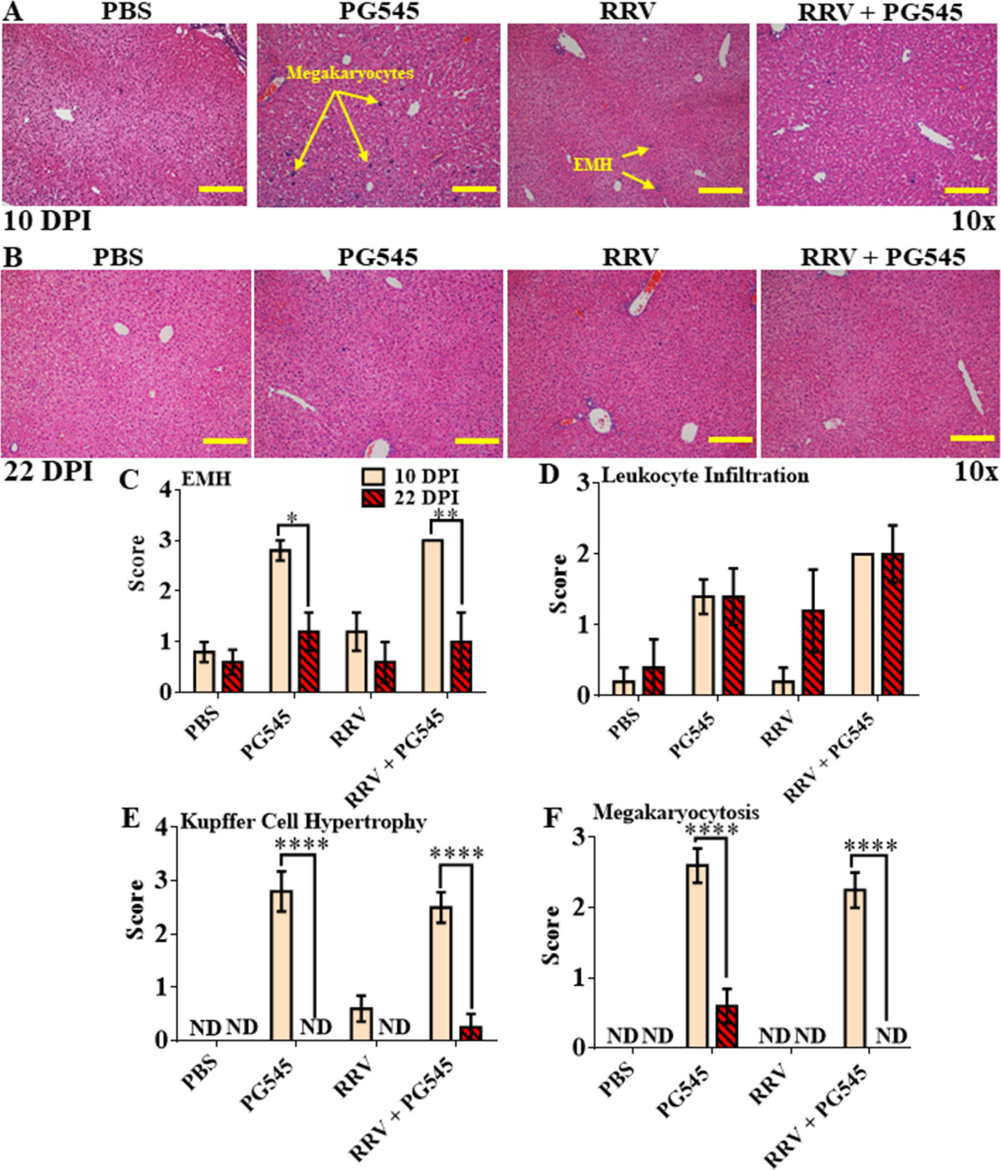
Prolonged PG545 treatment causes reversible EMH, Kupffer cell hypertrophy in mice by 22 dpi. 17–20-day-old C57BL/6 mice were infected s.c. with 10^4^ PFU RRV or PBS alone on day 0. Then infected mice were either mock treated with PBS or with PG545 on −1, 4 and 9 dpi and subsequently, sacrificed at 10 dpi and 22 dpi. (A-B) For histological analysis, liver tissues were paraffin embedded, sectioned and stained with H&E. (C-F) Liver sections were assessed and scored according to level of EMH, megakaryocytosis, leukocyte infiltration and Kupffer cell hypertrophy for both 10 dpi and 22 dpi time points. All data and images represent the mean results ± SEM for at least 5 mice per group (Scale bar; 10× = 200**μ**m). PBS, mock-infected PBS control; PG545, mock-infected PG545-treated; RRV, RRV-infected PBS-treated; RRV + PG545, RRV-infected PG545-treated.

Given the observed hepatomegaly, histological analysis was conducted to determine the source of the temporary cellular expansion. Histological analysis of the liver revealed that the prolonged regime caused EMH expansion (**** = P<0.*0001*), megakaryocytosis (**** = P<0.*0001*), Kupffer cell hypertrophy (**** = P<0.*0001*) indicative of cellular activation, and leukocyte infiltration (** = P<0.*01*) in the mock-PG545 and RRV-PG545 mice at 10 dpi (Fig 8A, 8C, 8D, 8E and 8F). By 22 dpi, EMH, Kupffer cell hypertrophy and leukocyte infiltration between all groups were similar, with residual megakaryocytosis (* = P<0.*05*) in the mock-PG545 mice (Fig 8B, 8C, 8D, 8E and 8F). This indicates that prolonged PG545 treatment induces reversible hepatic inflammation.

## Discussion

In the current study, we explored the consequences of prolonged treatment of PG545 in RRV-infected mice alongside, the recently published standard PG545 treatment for RRVD in mice. This was done, to determine if an increase in the treatment frequency could impact the duration and further reduce the acute clinical disease observed in the RRV-infected mice. Additionally, the occurrence of compound-induced lymphoid organ changes, if any, were also critically evaluated, to determine compound efficacy and tolerability. During this study, it was first noted that, the PG545 treatment to both mock- and RRV-infected mice groups caused a reduction in weight gain across the treated groups. Other comparable murine studies of PG545 administration have also reported weight losses in treated animals [30].

The spleen analysis showed that standard PG545 treatment to both RRV-infected and mock-infected mice induced mild red pulp-based splenomegaly by means of EMH, while also causing a mild splenic white pulp atrophy with significant reduction in CD3+ T-lymphocytes, as demonstrated by the flow cytometry data. Additionally, PG545 treatment to RRV-infected mice also caused an enhanced granulocyte recruitment (vital in controlling virus infection) to the spleen parenchyma when compared to RRV-infected mock-treated mice, which supports the immunomodulatory function of PG545.

The liver analyses of the standard PG545 treatment revealed very mild EMH, megakaryocytosis and liver inflammation (Kupffer cell activation/hypertrophy, leukocyte infiltration). This therefore, indicates that the standard treatment may be a well-tolerated treatment for acute RRVD in mice. Further, hepatotoxicity was also evaluated by assaying specific tissue enzymes through serum biochemistry analyses. Although, AST and ALT can detect liver cell injury, these enzymes can also be elevated in non-hepatic conditions such as during acute-skeletal muscle injury such as rhabdomyolysis [31]. Interestingly, we had identified, RRV-mediated elevations of AST and ALT for the first time, in the serum of RRV-infected mock-treated group. Moreover, it is noteworthy that PG545 was able to return these elevated levels of AST and ALT to the baseline levels, post-treatment at 10 dpi. Both ALP and GLDH levels were also reduced in PG545-treated mice when compared to mock-PBS mice. Although, AST is not liver specific, GLDH is a specific marker of liver disease in all mammalian species. Thus, the reduced activity of GLDH in treated mice, further confirms the lack of hepatotoxicity in the standard PG545 treatment regimen for RRVD [32].

Interestingly, although the standard PG545 treatment reduced the elevations of ALT and AST enzymes, the treatment could not reduce the elevated creatinine levels that were also reported in the RRV-infected mock-treated mice for the first time. This may indicate the presence of a long-term RRV-mediated kidney sequalae present in RRV-infected mock-treated mice, that were not responsive to the PG545 treatment (S3 Fig). It was also noted that albumin was reduced in the serum of PG545-treated mock- and RRV-infected mouse cohorts. Studies have reported that serum albumin could be reduced by inflammation [33], and given that PG545 has been reported as an immunomodulatory compound, it is possible that serum levels of albumin may have been reduced as a result in the PG545-treated mock- and RRV-infected mice.

It was also noted that when PG545-treatment was administered for a prolonged duration, it caused a moderate to pronounced hepatosplenomegaly, in addition to adversely affecting the haematopoietic system by causing anaemia (haemoglobin <100mg/L) and the dysregulation of the red pulp /white pulp ratios within the spleens of treated mice. Drug-induced changes in lymphoid organ architecture and overall erythrocyte injury have also been previously reported with other anti-viral drugs [34]. For example, a study demonstrated that the anti-viral drug, ribavirin (RBV) administered for the treatment of chronic hepatitis C virus (HCV) infection, also caused a mild reversible haemolytic anaemia [34]. The study demonstrated that RBV treatment caused oxidative damage to the RBC membrane and the binding of specific IgG to aggregated band three, resulting in the early removal of damaged RBCs [34]. It is possible that a similar mechanism could have also occurred in the prolonged PG545-treatment regime. Hence, when the drug was in the systemic circulation, the spleen could have recognised the drug-coated erythrocyte membranes to be non-self and destroyed the cells without recycling the haemoglobin back into the peripheral circulation [35]. The increased lysis of red blood cells, at a rate faster than erythropoiesis, may have further resulted in secondary anaemia as well as the pronounced splenomegaly. While some level of EMH is always present in all rodents regardless of health status, EMH is often increased when the body requires red or white blood cells physiologically and, moreover, splenic erythropoiesis is frequently positively correlated with anaemia regardless of the aetiology of haemolysis [36].

Other studies also reveal that PG545 has the capacity to significantly decrease cell proliferation, while also increasing apoptosis, especially in tumour tissues [15]. We noted both red and white compartment-dependent increases and decreases in cellular proliferation of the splenocytes following the prolonged, PG545 treatment. For example, there was a massive expansion and proliferation of erythroid and myeloid cells, which were supported by increases in expression levels of TfR1, HBB-1, GATA-1 and CSF2 transcripts within the red pulp, while the white pulp underwent mild to moderate atrophy, reflected by the reduced expression levels of CD40LG, CD8α+, CD4+ and IKZF1 transcripts. CD40LG is expressed on the surface of T-cells and is a regulator of B-cell function. Transcription factor IKZF1 is an important regulator of lymphoid differentiation that is often upregulated in the presence of B-cells, CD4+ and CD8a+ T-cells, and NK cells, while it is also conversely down-regulated in erythroblasts, megakaryocytes and monocytes [37–39]. This was also consistent with the erythroblast, megakaryocyte and monocyte expansion detected in the histological analysis of spleens from PG545-treated mock and infected mice. Moreover, it was also found that RRV-infected mock-treated mice underwent systemic lymphocytosis in addition to having hypertrophic white pulps, presumably in response to viral antigens.

TfR1 are vital for the development of erythrocytes and are expressed on the cell surface of erythroid precursors while HBB-1 associates with haemoglobin, subunit alpha 1 (HBA-1) to form, haemoglobin A that is present in red blood cells [40, 41]. GATA-1 is a transcriptional modulator (activator or repressor) for erythroid development, differentiation and often found upregulated in splenomegaly [42]. Thus, the upregulation of TfR1, HBA1 and GATA-1 in the mock-PG545 and RRV-infected PG545-treated groups suggests that the spleens were undergoing synthesis of new red blood cells or an expansion of the red pulp. CSF2 stimulates stem cells to produce granulocytes and monocytes where, monocytes can migrate to tissues and differentiate into macrophages and dendritic cells and drive PG545 to participate in immunomodulatory functions as mentioned above [19, 43]. A majority of pre-clinical and clinical data suggest that IL-10 is a potent and significant anti-inflammatory host factor. IL-10 can impede the synthesis of pro-inflammatory cytokines, such as interferon gamma (IFN-γ) and tumour necrosis factor (TNF-α) by Th1 T-cells and macrophage, in addition to supressing antigen-presenting cells [44, 45]. However, IL-10 can also be pro-stimulatory towards Th2 T-cells, mast cells, B-cell maturation and antibody production. In our study as we noted a reduction in IFN-γ in RRV-PG545 mice [12], thus it is most likely that IL-10 is performing as an anti-inflammatory host factor with regards to the PG545 treatment. However, it should be noted that these increases and decreases in the expression levels of the haemopoietic influences were reversible and no longer present in the spleens by 22 dpi.

Additionally, when PG545 dosage was increased from the standard two to the prolonged three doses, a dose-related hepatomegaly was noted. The initial absence of drug induced toxicity at dual doses was evidence that the hepatomegaly was likely an adaptation to a heightened functional load in the liver due to drug processing (detoxification, filtration of toxic metabolites and the removal of damaged erythrocytes) [46]. Furthermore, haemolytic anaemia may have also caused the hepatomegaly due to increased EMH and Kupffer cell activity. Other studies have shown that in general drug-induced toxicity may include biological events such as haemolytic anaemia, hepatic toxicity or splenomegaly [47–51].

PG545 has a half-life of approximately 141 hours (6 days) in humans and more than 50 hours (2 days) in mice [13, 16, 52]. Thus, the increased frequency of treatment doses could have also been a factor in causing toxicity in the mice that were treated under the prolonged treatment regime. Importantly, we noted that cessation of PG545 treatment allowed mice to recover from the drug induced hepatosplenomegaly and anaemia as seen at 22 dpi (approximately 2 weeks post last treatment). Consistent with our results, other PG545 studies report similar PG545-induced side effects as transient [52].

In summary, this paper provides encouraging results for the use of PG545 as an anti-inflammatory and an anti-viral treatment. When administered at lower doses, our data suggests that PG545 has the potential to alleviate RRVD with very minor drug induced organ pathology. Although, high doses of PG545 caused moderate histopathological and biological events such as anaemia and related hepatosplenomegaly, these were short-lived and reversible effects with a quick return to baseline levels when treatment was terminated.

## Supporting information

S1 Fig Dosing schedule of PG545 in mice and the percentages of weight gain in mice. (A) The timeline of the dosing schedules of PG545 treatment in mice. (B, C) In the standard treatment regimen, 17-20-day-old C57BL/6 mice were infected s.c. with 10^4^ PFU/50 μl RRV or PBS alone on day 0 in the thorax and received s.c. injections of PG545 or mock-treated with PBS on -1 and 4 dpi. (D, E) In prolonged treatment dose, C57BL/6 mice were infected (s.c.) with 10^4^ PFU/50 μl RRV or PBS alone on day 0 and received s.c. injections of PG545 or PBS diluents from −1, 4 and 9 dpi. Mice were also monitored till 22 dpi at disease resolution.
(TIF)

S2 Fig Standard PG545-treated liver histology of mice at 10 dpi. 17–20-day-old C57BL/6 mice were infected s.c. with 10^4^ PFU RRV or mock treated with PBS or with PG545 on −1 and 4 dpi. Mice were culled at 10 dpi and liver was harvested. Liver sections were stained with H&E. Images are representative images for at least 5 mice per group. (Scale bar; 10x = 200**μ**m). PBS, mock-infected PBS control; PG545, mock-infected PG545-treated; RRV, RRV-infected PBS-treated; RRV + PG545, RRV-infected PG545-treated.
(TIF)

S3 Fig Standard PG545-treated kidney histology and tubular pathology scores of mice at 10 dpi. 17–20-day-old C57BL/6 mice were infected s.c. with 10^4^ PFU RRV or mock treated with PBS or with PG545 on −1 and 4 dpi. Mice were culled at 10 dpi and kidneys were harvested. (A) Kidney sections were stained with H&E and (B) scored for the presence of tubular injury. The proximal tubules in the kidneys of both the RRV and RRV + PG545 treated mice were found to be mildly to moderately dilated with mild attenuation of the epithelial cells, in addition to the presence of increased protein-rich material within the tubular lumen of the kidneys. Images are representative images for at least 5 mice per group. (Scale bar; 20x = 150**μ**m). PBS, mock-infected PBS control; PG545, mock-infected PG545-treated; RRV, RRV-infected PBS-treated; RRV + PG545, RRV-infected PG545-treated.
(TIF)

S4 Fig Heat map representing color -coded expression levels of differentially expressed genes (red; up-regulation, green: down-regulation) in prolonged PG545 treated, spleen tissues. 17-20-day-old C57BL/6 mice were infected (s.c.) with 10^4^ PFU/50 **μ**l RRV or PBS alone on day 0 and received s.c. injections of PG545 or PBS diluents from −1, 4 and 9 dpi. Mice were sacrificed on 10 dpi and spleens were homogenised and RNA extracted to analyse haemopoietic influencing soluble host factors, transcription factors or immunocyte expression via RT-qPCR using a commercial pre-plated plate.
(TIF)
