# Supplementary figures and images for: PG545 treatment reduces RRV-induced elevations of AST, ALT with secondary lymphoid organ alterations in C57BL/6 mice
Source: PLoS One. 2019 Jun 6;14(6):e0217998. doi: 10.1371/journal.pone.0217998 (PMC6553857; doi:10.1371/journal.pone.0217998)

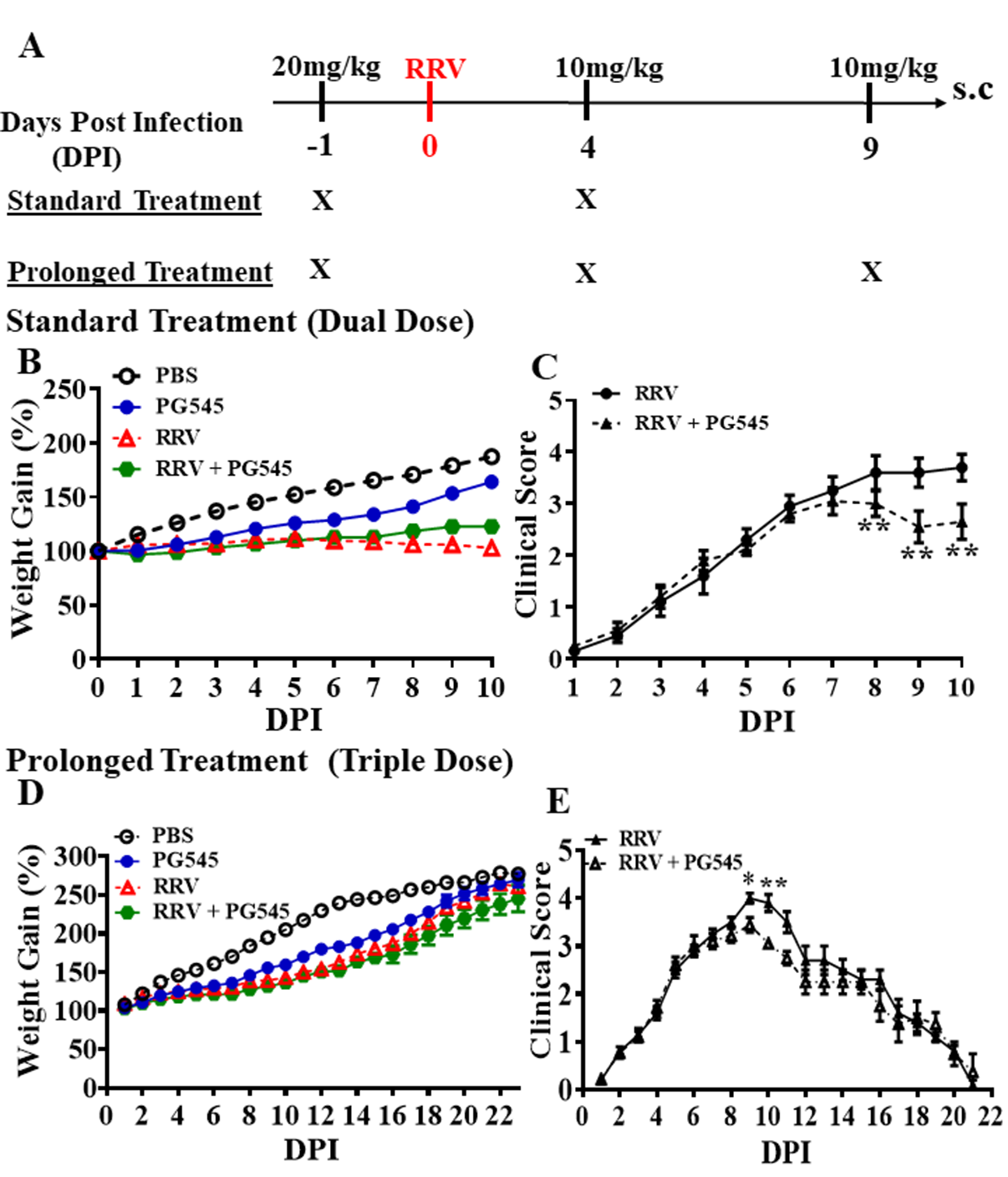

Supplement: S1 Fig — (A) The timeline of the dosing schedules of PG545 treatment in mice. (B, C) In the standard treatment regimen, 17-20-day-old C57BL/6 mice were infected s.c. with 104 PFU/50 μl RRV or PBS alone on day 0 in the thorax and received s.c. injections of PG545 or mock-treated with PBS on -1 and 4 dpi. (D, E) In prolonged treatment dose, C57BL/6 mice were infected (s.c.) with 104 PFU/50 μl RRV or PBS alone on day 0 and received s.c. injections of PG545 or PBS diluents from −1, 4 and 9 dpi. Mice were also monitored till 22 dpi at disease resolution. (TIF) [file pone.0217998.s001.tif]

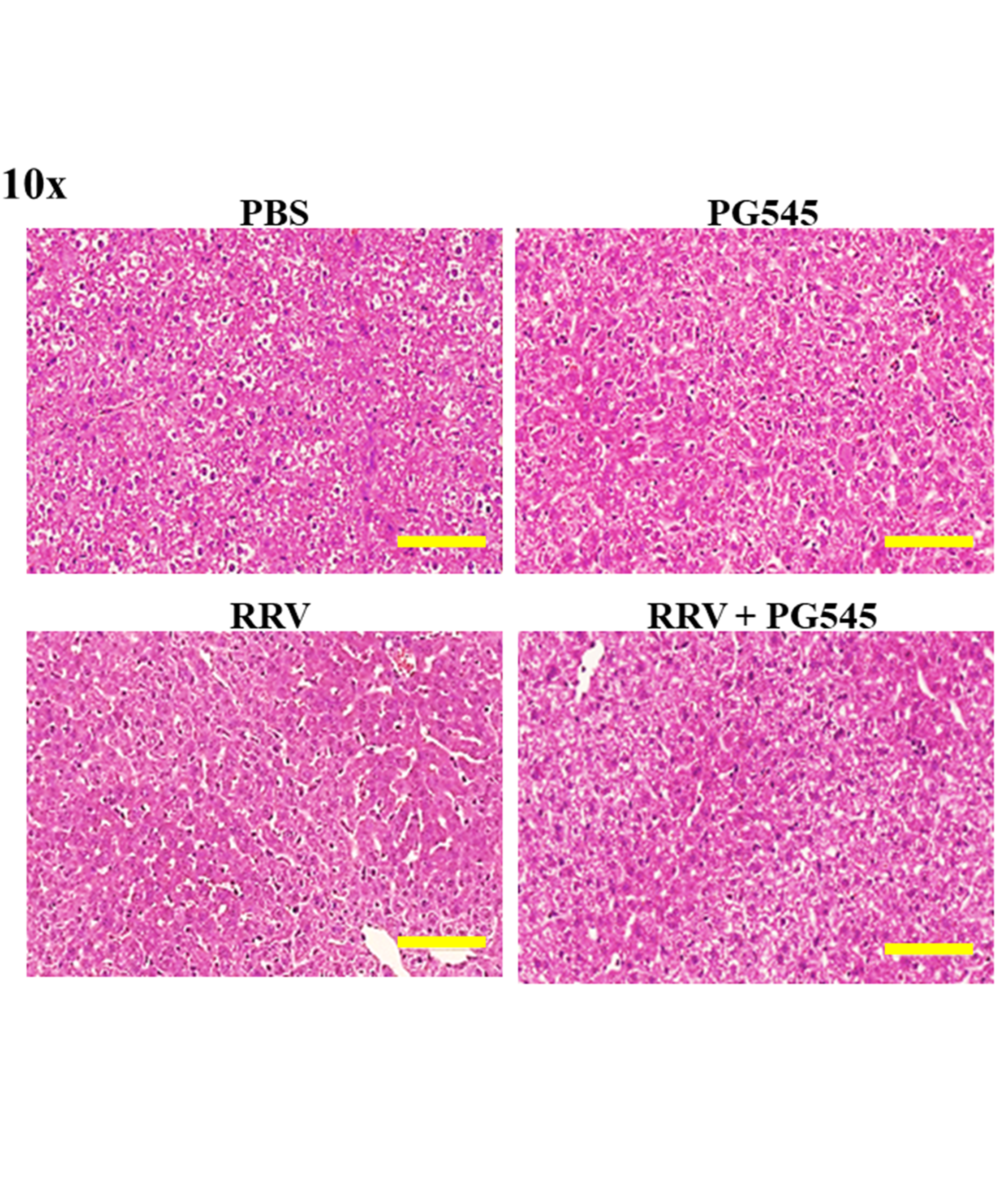

Supplement: S2 Fig — 17–20-day-old C57BL/6 mice were infected s.c. with 104 PFU RRV or mock treated with PBS or with PG545 on −1 and 4 dpi. Mice were culled at 10 dpi and liver was harvested. Liver sections were stained with H&E. Images are representative images for at least 5 mice per group. (Scale bar; 10x = 200μm). PBS, mock-infected PBS control; PG545, mock-infected PG545-treated; RRV, RRV-infected PBS-treated; RRV + PG545, RRV-infected PG545-treated. (TIF) [file pone.0217998.s002.tif]

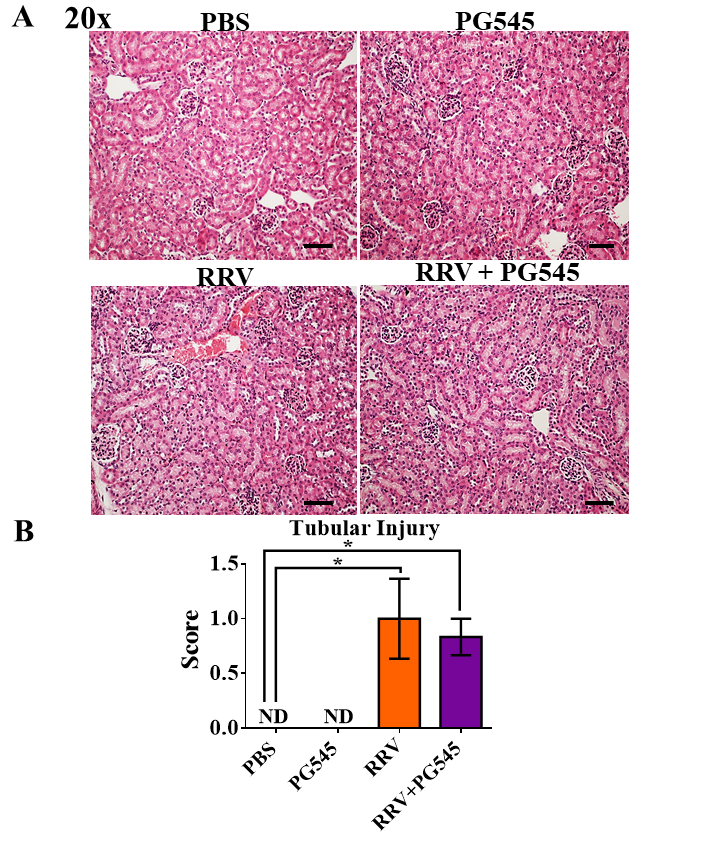

Supplement: S3 Fig — 17–20-day-old C57BL/6 mice were infected s.c. with 104 PFU RRV or mock treated with PBS or with PG545 on −1 and 4 dpi. Mice were culled at 10 dpi and kidneys were harvested. (A) Kidney sections were stained with H&E and (B) scored for the presence of tubular injury. The proximal tubules in the kidneys of both the RRV and RRV + PG545 treated mice were found to be mildly to moderately dilated with mild attenuation of the epithelial cells, in addition to the presence of increased protein-rich material within the tubular lumen of the kidneys. Images are representative images for at least 5 mice per group. (Scale bar; 20x = 150μm). PBS, mock-infected PBS control; PG545, mock-infected PG545-treated; RRV, RRV-infected PBS-treated; RRV + PG545, RRV-infected PG545-treated. (TIF) [file pone.0217998.s003.tif]

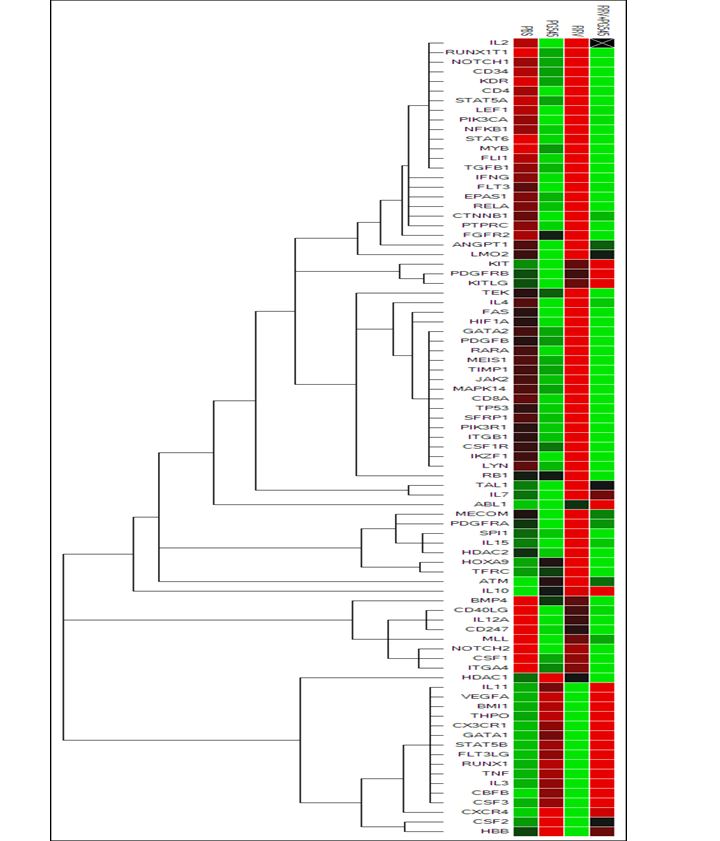

Supplement: S4 Fig — 17-20-day-old C57BL/6 mice were infected (s.c.) with 104 PFU/50 μl RRV or PBS alone on day 0 and received s.c. injections of PG545 or PBS diluents from −1, 4 and 9 dpi. Mice were sacrificed on 10 dpi and spleens were homogenised and RNA extracted to analyse haemopoietic influencing soluble host factors, transcription factors or immunocyte expression via RT-qPCR using a commercial pre-plated plate. (TIF) [file pone.0217998.s004.tif]
